# {4,4′-Dichloro-2,2′-[2,2-dimethyl­propane-1,3-diylbis(nitrilo­methanylyl­idene)]diphenolato}copper(II)

**DOI:** 10.1107/S1600536812033491

**Published:** 2012-07-28

**Authors:** Hadi Kargar, Reza Kia, Fatemeh Ganji, Valiollah Mirkhani

**Affiliations:** a Department of Chemistry, Payame Noor University, PO Box 19395-3697 Tehran, I. R. of IRAN; b Department of Chemistry, Science and Research Branch, Islamic Azad University, Tehran, Iran; c Department of Chemistry, University of Isfahan, 81746-73441, Isfahan, Iran

## Abstract

In the title Schiff base complex, [Cu(C_19_H_18_Cl_2_N_2_O_2_)], the Cu^II^ ion is coordinated in a distorted square-planar environment by two N atoms and two O atoms of the tetra­dentate ligand. The dihedral angle between the benzene rings is 36.86 (14)°. In the crystal, mol­ecules are linked into inversion dimers by pairs of weak C—H⋯O hydrogen bonds. In addition, π–π [centroid–centroid distance = 3.7279 (16) Å] and weak C—H⋯π inter­actions are observed.

## Related literature

For applications of Schiff bases in coordination chemistry, see: Granovski *et al.* (1993 ▶); Blower *et al.* (1998 ▶). For related structures, see: Ghaemi *et al.* (2011 ▶); Kargar *et al.* (2011 ▶, 2012 ▶). For standard bond lengths, see: Allen *et al.* (1987 ▶).
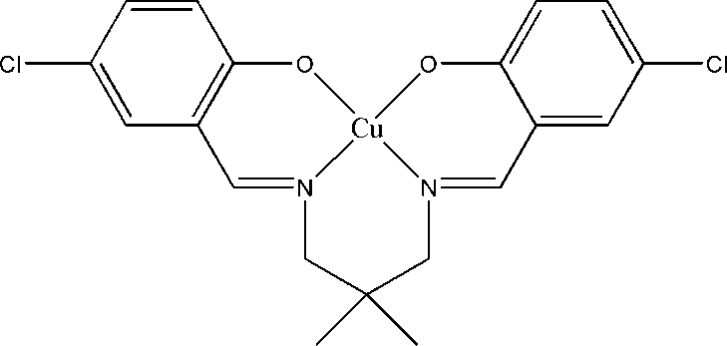


## Experimental

### 

#### Crystal data

[Cu(C_19_H_18_Cl_2_N_2_O_2_)]
*M* *_r_* = 440.79
Triclinic, 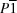

*a* = 9.4213 (12) Å
*b* = 9.5718 (13) Å
*c* = 11.4392 (15) Å
α = 74.478 (10)°
β = 78.635 (10)°
γ = 73.339 (10)°
*V* = 944.1 (2) Å^3^
*Z* = 2
Mo *K*α radiation
μ = 1.46 mm^−1^
*T* = 296 K
0.23 × 0.12 × 0.08 mm

#### Data collection

Bruker SMART APEXII CCD area-detector diffractometer
Absorption correction: multi-scan (*SADABS*; Bruker, 2005 ▶) *T*_min_ = 0.731, *T*_max_ = 0.893
8620 measured reflections
4302 independent reflections
3369 reflections with *I* > 2σ(*I*)
*R*_int_ = 0.049

#### Refinement

*R*[*F* ^2^ > 2σ(*F* ^2^)] = 0.042
*wR*(*F* ^2^) = 0.098
*S* = 1.00
4302 reflections
236 parameters
H-atom parameters constrained
Δρ_max_ = 0.44 e Å^−3^
Δρ_min_ = −0.46 e Å^−3^

### 

Data collection: *APEX2* (Bruker, 2005 ▶); cell refinement: *SAINT* (Bruker, 2005 ▶); data reduction: *SAINT*; program(s) used to solve structure: *SHELXTL* (Sheldrick, 2008 ▶); program(s) used to refine structure: *SHELXTL*; molecular graphics: *SHELXTL*; software used to prepare material for publication: *SHELXTL* and *PLATON* (Spek, 2009 ▶).

## Supplementary Material

Crystal structure: contains datablock(s) global, I. DOI: 10.1107/S1600536812033491/lh5504sup1.cif

Structure factors: contains datablock(s) I. DOI: 10.1107/S1600536812033491/lh5504Isup2.hkl

Additional supplementary materials:  crystallographic information; 3D view; checkCIF report

## Figures and Tables

**Table 1:** Hydrogen-bond geometry (Å, °) *Cg* is centroid of Cu1/O2/C17/C12/C11/N2.

*D*—H⋯*A*	*D*—H	H⋯*A*	*D*⋯*A*	*D*—H⋯*A*
C16—H16*A*⋯O2^i^	0.93	2.46	3.367 (3)	165
C10—H10*B*⋯*Cg* ^ii^	0.97	2.65	3.452 (3)	140

Symmetry codes: (i) 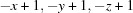; (ii) 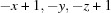.

**Fig. 1.**
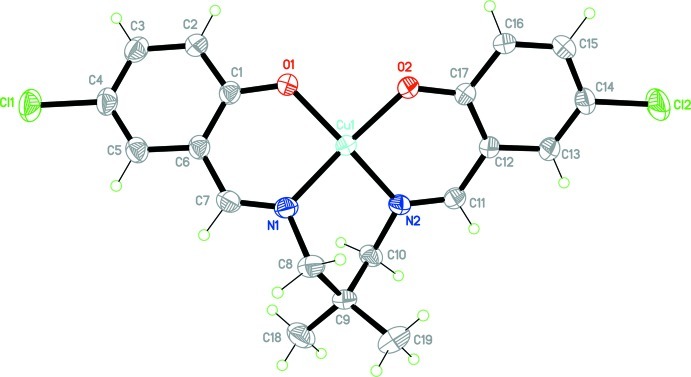
The molecular structure of the title compound, showing 40% probability displacement ellipsoids and the atomic numbering.

**Fig. 2.**
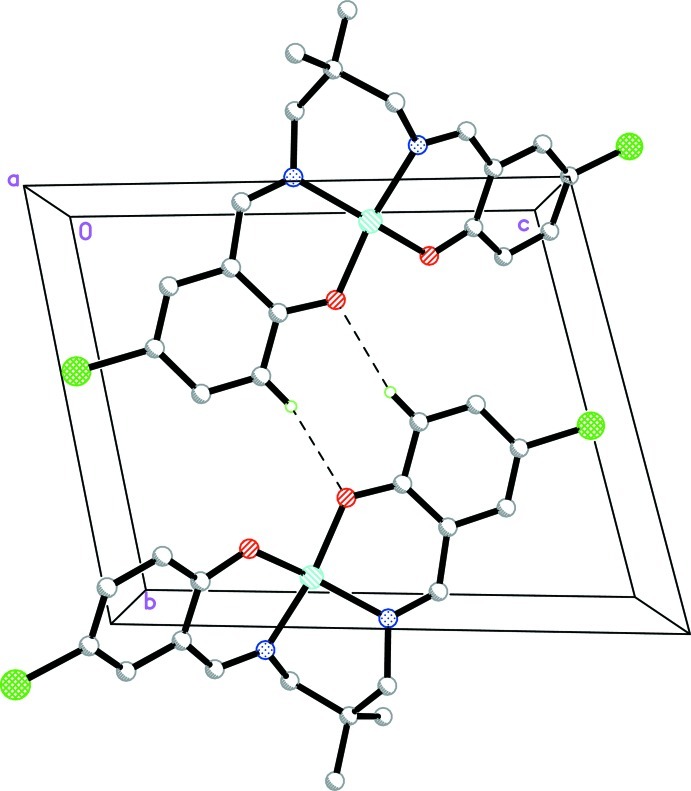
A part of the crystal structure of the title compound showing dimer formation through weak intermolecular C—H···O hydrogen bonds (dashed lines). Only the H atoms involved in hydrogen bonds are shown.

## supplementary crystallographic information

### Comment

Schiff base complexes are one of the most important stereochemical models in transition metal coordination chemistry, with ease of preparation and structural variations (Granovski *et al.,* 1993; Blower *et al.,* (1998). In continuation of our work on the crystal structure of Schiff base metal complexes (Kargar *et al.,* 2012; Kargar *et al.,* 2011; Ghaemi, *et al.,* (2011), we have determined the X-ray structure of the title compound.

The asymmetric unit of the title compound, Fig. 1, comprises a Schiff base complex. The bond lengths (Allen *et al.,* 1987) and angles are within the normal ranges and are comparable to those in related structures (Kargar *et al.,* 2012; Kargar *et al.,* 2011; Ghaemi, *et al.,* (2011).

The coordination geometry of the Cu^II^ ion is distorted square-planar which is supported by the N_2_O_2_ donor atoms of the coordinated Schiff base ligand. The dihedral angle between the substituted benzene rings is 36.86 (14)°. In the crystal, molecules are linked by a pair of weak C—H···O hydrogen bonds, forming inversion dimers (Table 1, Fig. 2). The crystal structure is further stabilized by intermolecular π–π interactions [*Cg*1···*Cg*2^iii^ = 3.7279 (16)Å; (iii) 1 - *x*, -*y*, 2 - *z*; *Cg*1 and *Cg*2 are centroids of the Cu1/O1/C1/C6/C7/N1 and C1–C6 rings] and C—H···π interactions (Table 1).

### Experimental

The title compound was synthesized by adding 5-dichloro-salicylaldehyde-2,2-dimethyl-1, 3-propanediamine (2 mmol) to a solution of CuCl_2_. 4H_2_O (2.1 mmol) in ethanol (30 ml). The mixture was refluxed with stirring for half an hour. The resultant solution was filtered. Dark-green single crystals of the title compound suitable for *X*-ray structure determination were recrystallized from ethanol by slow evaporation of the solvents at room temperature over several days.

### Refinement

The H-atoms were included in calculated positions and treated as riding atoms: C—H = 0.93, 0.96 and 0.97 Å for CH, CH_3_ and CH_2_ H-atoms, respectively, with U_iso_ (H) = k × U_eq_(C), where k = 1.5 for CH_3_ H-atoms, and k = 1.2 for all other H-atoms.

### Crystal data

**Table:**

[Cu(C_19_H_18_Cl_2_N_2_O_2_)]	*Z* = 2
*M**_r_* = 440.79	*F*(000) = 450
Triclinic, *P*1	*D*_x_ = 1.551 Mg m^−^^3^
Hall symbol: -P 1	Mo *K*α radiation, λ = 0.71073 Å
*a* = 9.4213 (12) Å	Cell parameters from 1540 reflections
*b* = 9.5718 (13) Å	θ = 2.5–27.4°
*c* = 11.4392 (15) Å	µ = 1.46 mm^−^^1^
α = 74.478 (10)°	*T* = 296 K
β = 78.635 (10)°	Block, dark-green
γ = 73.339 (10)°	0.23 × 0.12 × 0.08 mm
*V* = 944.1 (2) Å^3^	

### Data collection

**Table:**

Bruker SMART APEXII CCD area-detector diffractometer	4302 independent reflections
Radiation source: fine-focus sealed tube	3369 reflections with *I* > 2σ(*I*)
Graphite monochromator	*R*_int_ = 0.049
φ and ω scans	θ_max_ = 27.5°, θ_min_ = 1.9°
Absorption correction: multi-scan (*SADABS*; Bruker, 2005)	*h* = −12→12
*T*_min_ = 0.731, *T*_max_ = 0.893	*k* = −12→12
8620 measured reflections	*l* = −14→11

### Refinement

**Table:**

Refinement on *F*^2^	Secondary atom site location: difference Fourier map
Least-squares matrix: full	Hydrogen site location: inferred from neighbouring sites
*R*[*F*^2^ > 2σ(*F*^2^)] = 0.042	H-atom parameters constrained
*wR*(*F*^2^) = 0.098	*w* = 1/[σ^2^(*F*_o_^2^) + (0.0521*P*)^2^] where *P* = (*F*_o_^2^ + 2*F*_c_^2^)/3
*S* = 1.00	(Δ/σ)_max_ = 0.001
4302 reflections	Δρ_max_ = 0.44 e Å^−^^3^
236 parameters	Δρ_min_ = −0.46 e Å^−^^3^
0 restraints	Extinction correction: *SHELXTL* (Sheldrick, 2008), Fc^*^=kFc[1+0.001xFc^2^λ^3^/sin(2θ)]^-1/4^
Primary atom site location: structure-invariant direct methods	Extinction coefficient: 0.044 (2)

### Special details

**Table:**

Geometry. All esds (except the esd in the dihedral angle between two l.s. planes) are estimated using the full covariance matrix. The cell esds are taken into account individually in the estimation of esds in distances, angles and torsion angles; correlations between esds in cell parameters are only used when they are defined by crystal symmetry. An approximate (isotropic) treatment of cell esds is used for estimating esds involving l.s. planes.
Refinement. Refinement of F^2^ against ALL reflections. The weighted R-factor wR and goodness of fit S are based on F^2^, conventional R-factors R are based on F, with F set to zero for negative F^2^. The threshold expression of F^2^ > 2sigma(F^2^) is used only for calculating R-factors(gt) etc. and is not relevant to the choice of reflections for refinement. R-factors based on F^2^ are statistically about twice as large as those based on F, and R- factors based on ALL data will be even larger.

### Fractional atomic coordinates and isotropic or equivalent isotropic displacement parameters (Å^2^)

**Table:**

	*x*	*y*	*z*	*U*_iso_*/*U*_eq_	
Cu1	0.59520 (4)	0.06692 (3)	0.63656 (3)	0.03281 (12)	
Cl1	0.14886 (11)	−0.15287 (10)	1.21334 (8)	0.0638 (2)	
Cl2	0.81316 (14)	0.43389 (11)	0.01358 (8)	0.0801 (3)	
O1	0.4216 (2)	0.1436 (2)	0.74001 (17)	0.0395 (4)	
O2	0.5916 (2)	0.2592 (2)	0.53387 (17)	0.0402 (4)	
N1	0.6589 (2)	−0.1147 (2)	0.7608 (2)	0.0345 (5)	
N2	0.6982 (2)	−0.0370 (2)	0.50664 (19)	0.0323 (5)	
C1	0.3645 (3)	0.0694 (3)	0.8440 (2)	0.0336 (5)	
C2	0.2211 (3)	0.1361 (3)	0.8969 (3)	0.0392 (6)	
H2A	0.1697	0.2291	0.8560	0.047*	
C3	0.1548 (3)	0.0676 (3)	1.0075 (3)	0.0439 (7)	
H3A	0.0600	0.1141	1.0401	0.053*	
C4	0.2299 (3)	−0.0707 (3)	1.0697 (3)	0.0427 (7)	
C5	0.3678 (3)	−0.1413 (3)	1.0215 (2)	0.0401 (6)	
H5A	0.4166	−0.2344	1.0641	0.048*	
C6	0.4369 (3)	−0.0746 (3)	0.9079 (2)	0.0341 (5)	
C7	0.5839 (3)	−0.1549 (3)	0.8646 (2)	0.0367 (6)	
H7A	0.6278	−0.2433	0.9163	0.044*	
C8	0.8107 (3)	−0.2035 (3)	0.7313 (3)	0.0391 (6)	
H8A	0.8418	−0.2764	0.8046	0.047*	
H8B	0.8782	−0.1380	0.7059	0.047*	
C9	0.8246 (3)	−0.2868 (3)	0.6290 (3)	0.0370 (6)	
C10	0.7130 (3)	−0.1996 (3)	0.5388 (2)	0.0365 (6)	
H10A	0.7437	−0.2370	0.4645	0.044*	
H10B	0.6158	−0.2182	0.5739	0.044*	
C11	0.7392 (3)	0.0260 (3)	0.3960 (2)	0.0326 (5)	
H11A	0.7864	−0.0363	0.3424	0.039*	
C12	0.7187 (3)	0.1840 (3)	0.3477 (2)	0.0319 (5)	
C13	0.7706 (3)	0.2307 (3)	0.2225 (2)	0.0388 (6)	
H13A	0.8194	0.1597	0.1766	0.047*	
C14	0.7498 (3)	0.3784 (3)	0.1688 (3)	0.0441 (7)	
C15	0.6757 (3)	0.4870 (3)	0.2359 (3)	0.0450 (7)	
H15A	0.6615	0.5880	0.1981	0.054*	
C16	0.6242 (3)	0.4443 (3)	0.3570 (3)	0.0406 (6)	
H16A	0.5745	0.5175	0.4005	0.049*	
C17	0.6445 (3)	0.2919 (3)	0.4180 (2)	0.0333 (5)	
C18	0.7882 (4)	−0.4381 (3)	0.6845 (3)	0.0563 (8)	
H18A	0.7969	−0.4890	0.6206	0.084*	
H18B	0.6880	−0.4233	0.7263	0.084*	
H18C	0.8568	−0.4973	0.7414	0.084*	
C19	0.9840 (4)	−0.3059 (4)	0.5632 (4)	0.0598 (9)	
H19A	0.9953	−0.3568	0.4989	0.090*	
H19B	1.0529	−0.3636	0.6203	0.090*	
H19C	1.0042	−0.2093	0.5290	0.090*	

### Atomic displacement parameters (Å^2^)

**Table:**

	*U*^11^	*U*^22^	*U*^33^	*U*^12^	*U*^13^	*U*^23^
Cu1	0.03676 (19)	0.02733 (17)	0.02913 (18)	−0.00348 (12)	0.00110 (12)	−0.00637 (12)
Cl1	0.0809 (6)	0.0601 (5)	0.0447 (4)	−0.0320 (5)	0.0197 (4)	−0.0069 (4)
Cl2	0.1256 (9)	0.0540 (5)	0.0398 (4)	−0.0221 (6)	0.0211 (5)	0.0012 (4)
O1	0.0420 (10)	0.0334 (9)	0.0324 (9)	−0.0021 (8)	0.0039 (8)	−0.0038 (8)
O2	0.0512 (11)	0.0292 (9)	0.0333 (10)	−0.0068 (8)	0.0072 (8)	−0.0083 (8)
N1	0.0364 (11)	0.0319 (11)	0.0326 (11)	−0.0007 (9)	−0.0056 (9)	−0.0103 (9)
N2	0.0372 (11)	0.0254 (10)	0.0335 (11)	−0.0062 (9)	−0.0034 (9)	−0.0074 (9)
C1	0.0388 (13)	0.0343 (13)	0.0295 (12)	−0.0111 (11)	−0.0020 (11)	−0.0099 (11)
C2	0.0383 (14)	0.0366 (14)	0.0392 (14)	−0.0071 (12)	0.0007 (12)	−0.0092 (12)
C3	0.0404 (15)	0.0474 (16)	0.0446 (16)	−0.0147 (13)	0.0069 (13)	−0.0165 (14)
C4	0.0553 (17)	0.0431 (15)	0.0338 (14)	−0.0247 (14)	0.0059 (13)	−0.0103 (12)
C5	0.0530 (16)	0.0358 (14)	0.0337 (14)	−0.0167 (13)	−0.0044 (12)	−0.0064 (11)
C6	0.0408 (14)	0.0342 (13)	0.0294 (12)	−0.0124 (11)	−0.0030 (11)	−0.0083 (11)
C7	0.0449 (15)	0.0296 (12)	0.0344 (13)	−0.0042 (11)	−0.0110 (12)	−0.0065 (11)
C8	0.0360 (14)	0.0376 (14)	0.0424 (15)	−0.0007 (11)	−0.0089 (12)	−0.0128 (12)
C9	0.0375 (14)	0.0280 (12)	0.0416 (15)	−0.0014 (11)	−0.0019 (12)	−0.0106 (11)
C10	0.0464 (15)	0.0259 (12)	0.0383 (14)	−0.0102 (11)	−0.0068 (12)	−0.0071 (11)
C11	0.0346 (13)	0.0327 (12)	0.0311 (13)	−0.0070 (10)	−0.0021 (10)	−0.0114 (11)
C12	0.0323 (12)	0.0306 (12)	0.0312 (13)	−0.0085 (10)	−0.0004 (10)	−0.0065 (10)
C13	0.0440 (15)	0.0368 (14)	0.0328 (13)	−0.0098 (12)	0.0044 (12)	−0.0101 (11)
C14	0.0536 (17)	0.0414 (15)	0.0319 (14)	−0.0149 (13)	0.0013 (13)	−0.0009 (12)
C15	0.0501 (16)	0.0305 (13)	0.0462 (16)	−0.0087 (12)	−0.0009 (13)	−0.0002 (12)
C16	0.0419 (14)	0.0291 (13)	0.0425 (15)	−0.0037 (11)	0.0059 (12)	−0.0084 (11)
C17	0.0304 (12)	0.0327 (12)	0.0336 (13)	−0.0066 (10)	0.0005 (10)	−0.0067 (11)
C18	0.081 (2)	0.0308 (14)	0.0511 (18)	−0.0071 (15)	−0.0115 (17)	−0.0047 (13)
C19	0.0419 (17)	0.066 (2)	0.068 (2)	−0.0013 (16)	0.0039 (16)	−0.0295 (19)

### Geometric parameters (Å, º)

**Table:**

Cu1—O2	1.8952 (18)	C8—H8A	0.9700
Cu1—O1	1.9050 (18)	C8—H8B	0.9700
Cu1—N2	1.952 (2)	C9—C18	1.525 (4)
Cu1—N1	1.953 (2)	C9—C19	1.525 (4)
Cl1—C4	1.749 (3)	C9—C10	1.527 (4)
Cl2—C14	1.747 (3)	C10—H10A	0.9700
O1—C1	1.312 (3)	C10—H10B	0.9700
O2—C17	1.308 (3)	C11—C12	1.434 (3)
N1—C7	1.280 (3)	C11—H11A	0.9300
N1—C8	1.466 (3)	C12—C13	1.413 (4)
N2—C11	1.283 (3)	C12—C17	1.418 (4)
N2—C10	1.471 (3)	C13—C14	1.355 (4)
C1—C2	1.411 (4)	C13—H13A	0.9300
C1—C6	1.421 (4)	C14—C15	1.398 (4)
C2—C3	1.378 (4)	C15—C16	1.367 (4)
C2—H2A	0.9300	C15—H15A	0.9300
C3—C4	1.385 (4)	C16—C17	1.414 (4)
C3—H3A	0.9300	C16—H16A	0.9300
C4—C5	1.365 (4)	C18—H18A	0.9600
C5—C6	1.407 (4)	C18—H18B	0.9600
C5—H5A	0.9300	C18—H18C	0.9600
C6—C7	1.442 (4)	C19—H19A	0.9600
C7—H7A	0.9300	C19—H19B	0.9600
C8—C9	1.550 (4)	C19—H19C	0.9600
			
O2—Cu1—O1	92.08 (8)	C19—C9—C10	110.3 (3)
O2—Cu1—N2	93.57 (8)	C18—C9—C8	109.9 (2)
O1—Cu1—N2	152.81 (9)	C19—C9—C8	107.9 (2)
O2—Cu1—N1	159.03 (9)	C10—C9—C8	111.1 (2)
O1—Cu1—N1	93.46 (9)	N2—C10—C9	114.0 (2)
N2—Cu1—N1	90.69 (9)	N2—C10—H10A	108.7
C1—O1—Cu1	126.44 (16)	C9—C10—H10A	108.7
C17—O2—Cu1	127.77 (16)	N2—C10—H10B	108.7
C7—N1—C8	119.4 (2)	C9—C10—H10B	108.7
C7—N1—Cu1	125.79 (18)	H10A—C10—H10B	107.6
C8—N1—Cu1	114.63 (18)	N2—C11—C12	125.8 (2)
C11—N2—C10	119.1 (2)	N2—C11—H11A	117.1
C11—N2—Cu1	125.51 (17)	C12—C11—H11A	117.1
C10—N2—Cu1	115.01 (17)	C13—C12—C17	120.0 (2)
O1—C1—C2	118.3 (2)	C13—C12—C11	116.9 (2)
O1—C1—C6	124.7 (2)	C17—C12—C11	123.1 (2)
C2—C1—C6	117.0 (2)	C14—C13—C12	120.4 (2)
C3—C2—C1	122.0 (3)	C14—C13—H13A	119.8
C3—C2—H2A	119.0	C12—C13—H13A	119.8
C1—C2—H2A	119.0	C13—C14—C15	120.7 (3)
C2—C3—C4	119.7 (3)	C13—C14—Cl2	119.7 (2)
C2—C3—H3A	120.1	C15—C14—Cl2	119.6 (2)
C4—C3—H3A	120.1	C16—C15—C14	119.9 (3)
C5—C4—C3	120.6 (3)	C16—C15—H15A	120.1
C5—C4—Cl1	119.9 (2)	C14—C15—H15A	120.1
C3—C4—Cl1	119.5 (2)	C15—C16—C17	121.8 (2)
C4—C5—C6	120.7 (3)	C15—C16—H16A	119.1
C4—C5—H5A	119.6	C17—C16—H16A	119.1
C6—C5—H5A	119.6	O2—C17—C16	118.5 (2)
C5—C6—C1	119.9 (2)	O2—C17—C12	124.3 (2)
C5—C6—C7	117.3 (2)	C16—C17—C12	117.2 (2)
C1—C6—C7	122.8 (2)	C9—C18—H18A	109.5
N1—C7—C6	125.4 (2)	C9—C18—H18B	109.5
N1—C7—H7A	117.3	H18A—C18—H18B	109.5
C6—C7—H7A	117.3	C9—C18—H18C	109.5
N1—C8—C9	113.3 (2)	H18A—C18—H18C	109.5
N1—C8—H8A	108.9	H18B—C18—H18C	109.5
C9—C8—H8A	108.9	C9—C19—H19A	109.5
N1—C8—H8B	108.9	C9—C19—H19B	109.5
C9—C8—H8B	108.9	H19A—C19—H19B	109.5
H8A—C8—H8B	107.7	C9—C19—H19C	109.5
C18—C9—C19	111.0 (3)	H19A—C19—H19C	109.5
C18—C9—C10	106.7 (2)	H19B—C19—H19C	109.5
			
O2—Cu1—O1—C1	−172.8 (2)	C8—N1—C7—C6	176.8 (2)
N2—Cu1—O1—C1	85.3 (3)	Cu1—N1—C7—C6	1.2 (4)
N1—Cu1—O1—C1	−13.0 (2)	C5—C6—C7—N1	176.0 (2)
O1—Cu1—O2—C17	−153.3 (2)	C1—C6—C7—N1	−7.1 (4)
N2—Cu1—O2—C17	0.1 (2)	C7—N1—C8—C9	111.5 (3)
N1—Cu1—O2—C17	101.4 (3)	Cu1—N1—C8—C9	−72.4 (3)
O2—Cu1—N1—C7	112.0 (3)	N1—C8—C9—C18	−87.2 (3)
O1—Cu1—N1—C7	6.9 (2)	N1—C8—C9—C19	151.7 (3)
N2—Cu1—N1—C7	−146.2 (2)	N1—C8—C9—C10	30.7 (3)
O2—Cu1—N1—C8	−63.9 (3)	C11—N2—C10—C9	114.9 (3)
O1—Cu1—N1—C8	−168.88 (17)	Cu1—N2—C10—C9	−71.5 (3)
N2—Cu1—N1—C8	38.01 (18)	C18—C9—C10—N2	161.2 (2)
O2—Cu1—N2—C11	0.1 (2)	C19—C9—C10—N2	−78.2 (3)
O1—Cu1—N2—C11	101.7 (3)	C8—C9—C10—N2	41.4 (3)
N1—Cu1—N2—C11	−159.3 (2)	C10—N2—C11—C12	173.6 (2)
O2—Cu1—N2—C10	−172.92 (17)	Cu1—N2—C11—C12	0.8 (4)
O1—Cu1—N2—C10	−71.3 (3)	N2—C11—C12—C13	−179.3 (3)
N1—Cu1—N2—C10	27.62 (18)	N2—C11—C12—C17	−2.0 (4)
Cu1—O1—C1—C2	−168.66 (18)	C17—C12—C13—C14	−0.1 (4)
Cu1—O1—C1—C6	11.3 (4)	C11—C12—C13—C14	177.2 (3)
O1—C1—C2—C3	−178.2 (2)	C12—C13—C14—C15	−0.5 (5)
C6—C1—C2—C3	1.8 (4)	C12—C13—C14—Cl2	−179.4 (2)
C1—C2—C3—C4	0.2 (4)	C13—C14—C15—C16	0.3 (5)
C2—C3—C4—C5	−1.3 (4)	Cl2—C14—C15—C16	179.2 (2)
C2—C3—C4—Cl1	177.0 (2)	C14—C15—C16—C17	0.5 (5)
C3—C4—C5—C6	0.4 (4)	Cu1—O2—C17—C16	177.33 (19)
Cl1—C4—C5—C6	−177.9 (2)	Cu1—O2—C17—C12	−1.2 (4)
C4—C5—C6—C1	1.7 (4)	C15—C16—C17—O2	−179.7 (3)
C4—C5—C6—C7	178.7 (2)	C15—C16—C17—C12	−1.0 (4)
O1—C1—C6—C5	177.4 (2)	C13—C12—C17—O2	179.4 (2)
C2—C1—C6—C5	−2.7 (4)	C11—C12—C17—O2	2.2 (4)
O1—C1—C6—C7	0.5 (4)	C13—C12—C17—C16	0.8 (4)
C2—C1—C6—C7	−179.6 (2)	C11—C12—C17—C16	−176.3 (2)

### Hydrogen-bond geometry (Å, º)

Cg is centroid of Cu1/O2/C17/C12/C11/N2.

**Table:**

*D*—H···*A*	*D*—H	H···*A*	*D*···*A*	*D*—H···*A*
C16—H16*A*···O2^i^	0.93	2.46	3.367 (3)	165
C10—H10*B*···*Cg*^ii^	0.97	2.65	3.452 (3)	140

Symmetry codes: (i) −*x*+1, −*y*+1, −*z*+1; (ii) −*x*+1, −*y*, −*z*+1.
